# Dynamic regression forecasting of carbapenem-resistant *Klebsiella* spp. based on carbapenem consumption with observed data from 2020 to 2023 and projections up to 2029 in a tertiary hospital in Alexandria, Egypt

**DOI:** 10.1186/s12879-026-13168-y

**Published:** 2026-04-11

**Authors:** Ehab Elmongui, Adel Zaki, Amel Elsheredy, Asmaa Abd Elhameed

**Affiliations:** 1 Department of Biomedical Informatics and Medical statistics, Medical Research Institute, Alexandria University, Alexandria, Egypt; 2 Department of Microbiology, Medical Research Institute, Alexandria University, Alexandria, Egypt

**Keywords:** Carbapenem-resistant *Klebsiella* spp., Carbapenem consumption, Antimicrobial resistance (AMR), Antimicrobial stewardship (AMS), Time-series analysis, Forecasting, COVID-19 impact, Statistical modeling

## Abstract

**Background:**

Carbapenem-resistant *Klebsiella* spp. is a major global health threat. Overuse of carbapenem in healthcare settings, particularly ICUs, has driven the rise of these resistant bacteria. This study aimed to apply dynamic regression forecasting to predict future trends of carbapenem-resistant *Klebsiella* spp., with analysis of the temporal relationship between carbapenem consumption and resistance serving as the foundation for building accurate forecasts to inform antimicrobial stewardship (AMS) strategies.

**Methods:**

This retrospective study analyzed hospital- and ICU-level carbapenem consumption and resistance data for 1,727 *Klebsiella* spp. isolates (1,043 ICU) from a tertiary care hospital in Alexandria, Egypt (2019–2023). Cross-correlation analyses identified lag structures between consumption and resistance, and these lagged consumption measures — hospital carbapenem use expressed as defined daily doses per 1,000 patient-days (DDD/1000PD) per quarter and ICU use expressed as days of therapy per 1,000 patient-days (DOT/1000PD) per semester —were incorporated as exogenous regressors in dynamic regression time-series models to forecast future resistance trends.

**Results:**

Carbapenem resistance exhibited by *Klebsiella* spp. in the hospital significantly increased from 30.2% (95% CI: 20.6% to 39.8%) in quarter 1 of 2020 to 57.5% (95% CI: 49.3% to 65.7%) in quarter 4 of 2023, accompanied by a parallel increase in the burden of resistant isolates and a substantial rise in carbapenem consumption from 30 DDD/1000PD in quarter 1 of 2019 to 131 DDD/1000PD in quarter 4 of 2023. Similarly, in the ICU, carbapenem resistance jumped from 25.8% (95% CI: 18.5% to 33.1%) in semester 1 of 2019 to 67.0% (95% CI: 58.6% to 75.3%) in semester 2 of 2023, accompanied by an increase in the burden of resistant isolates and by a surge in carbapenem consumption from 196 DOT/1000PD in semester 1 of 2019 to 473 DOT/1000PD in semester 4 of 2023. Strong correlations were found between one lagged period of carbapenem consumption and resistance rates (*r* = 0.80 for hospital, *r* = 0.86 for ICU). A marked increase in hospital carbapenem consumption was observed following the onset of the COVID-19 pandemic, with a post-pandemic level change of 61.82 DDD/1000 patient-days. Dynamic regression forecasting predicted stabilization of carbapenem resistance at approximately 50% at the hospital level (95% prediction interval [PI]: 32.6–67.1), while ICU-level models projected a continued upward trajectory in resistance over time (95% PI: 31.3–97.6).

**Conclusions:**

Dynamic regression forecasting revealed persistently high carbapenem resistance in *Klebsiella* spp. at the hospital level and a continued rising trend in the ICU, occurring in temporal association with carbapenem use. These projections support antimicrobial stewardship by informing trend awareness and highlighting the need for targeted interventions and sustained surveillance to mitigate further escalation.

**Supplementary Information:**

The online version contains supplementary material available at 10.1186/s12879-026-13168-y.

## Background

Antimicrobial resistance is escalating quickly across the globe, presenting a significant public health challenge that demands immediate and comprehensive action [1]. Carbapenem, as a last-resort antibiotic class, are indispensable in the management of severe infections caused by multidrug-resistant Gram-negative bacteria [2]. Nevertheless, their extensive use has unintentionally led to the rise and spread of carbapenem-resistant organisms (CRO), particularly *Klebsiella* species, which has complicated clinical treatments and increased mortality rates [3]. Since 2017, the WHO has identified carbapenem-resistant *Enterobacteriaceae* (including *Klebsiella* species) as one of the four highest-priority (critical) pathogens for antibiotic research and development [4].

Healthcare settings, especially intensive care units (ICUs), serve as hotspots for the acquisition and transmission of CRO due to the inherent vulnerability of critically ill patients, the pervasive use of invasive procedures and the extensive antimicrobial usage [5]. Therefore, understanding the dynamics between carbapenem consumption and the prevalence of carbapenem-resistant *Klebsiella* spp. is crucial for creating effective antimicrobial stewardship strategies and infection prevention and control measures.

Previous studies have underscored the strong association between carbapenem consumption and the prevalence of CRO. A study by Joseph NM et al. (2015) revealed a significant correlation between increased carbapenem use and the incidence of carbapenem-resistant *Klebsiella* spp. in a tertiary care teaching hospital [6]. Similarly, studies demonstrated that implementing restrictive carbapenem policies led to a reduction in Carbapenem resistance rates [7]. A recent study by H Elsawah et al. (2022) conducted in Egypt on *Klebsiella* species isolates in a tertiary care hospital revealed that introducing an antibiotic stewardship program in the ICU helped reduce unnecessary carbapenem usage and led to a lower incidence of *Klebsiella*-resistant strains [8]. These findings emphasize the pivotal role of antimicrobial stewardship in mitigating the spread of CRO.

However, in healthcare settings in Egypt, a critical knowledge gap remains in understanding both the temporal dynamics of carbapenem consumption and resistance, and the absence of forecasting studies that can anticipate future trends. While international studies have described associations, most relied on cross-sectional designs or short-term analyses, limiting their value for proactive intervention. To our knowledge, no local research has applied time-series forecasting techniques, particularly dynamic regression models, to antimicrobial resistance data in Egypt. Such approaches are essential for translating surveillance findings into actionable predictions that support antimicrobial stewardship.

This study addresses this gap by analyzing the temporal relationship between carbapenem consumption and carbapenem-resistant Klebsiella spp. over four years (2020–2023) in a tertiary care hospital in Alexandria, Egypt, and by applying dynamic regression forecasting to project resistance trends up to 2029. By integrating both aggregate hospital data and detailed ICU surveillance data, it provides healthcare decision makers with forward-looking evidence to guide stewardship strategies and public health planning.

## Methods

### Study design

This study was an observational retrospective study employing time series analysis to explore the temporal relationship between carbapenems’ consumption and percentage (%) resistance exhibited by *Klebsiella* spp. over a four-year period from January 2020 to December 2023. Both aggregate-level data from hospital records and infection control surveillance records in ICU were used to examine trends and associations between these variables over time.

### Study setting

The study was conducted at Gamal Abdel Naser Hospital, the main tertiary health insurance hospital in the Northwest region of Egypt. The hospital, located in Alexandria, serves the populations of Alexandria, El Beheira, and Marsa Matrouh governorates. With a total of 638 beds, including a 90-bed Intensive Care Unit (ICU) dedicated to critical care, the hospital provides comprehensive healthcare services to the region.

### Data collection

Data on carbapenems’ consumption and % resistance exhibited by *Klebsiella* spp. were collected and stored in Microsoft Excel spreadsheets. Data on carbapenem % resistance for the four years from Jan 2020 to Dec 2023 were retrieved for each of Imipenem, Meropenem, and Ertapenem from the WHONET database, on which the hospital depended for recording microbiologically and clinically documented infections. The hospital’s WHONET system was first established by the infection control administration in November 2019, capturing antimicrobial sensitivity results according to the Clinical and Laboratory Standards Institute (CLSI) breakpoints, starting from January 2020. Throughout the study period, antimicrobial susceptibility results were interpreted using a single CLSI standard (CLSI M100 Ed29, 2019), which was applied consistently from 2020 to 2023.

The total class % resistance to carbapenem was calculated as a weighted average, considering the number of times each class member was tested. For the hospital’s % carbapenem resistance, the number of isolates was sufficient to analyze data on quarterly basis while for ICU, the number of isolates allowed for biannual analysis (per semester).

The carbapenem consumption data collection included five years from Jan 2019 to Dec 2023 to allow studying the effect of lagged consumption on % resistance. The process involved gathering the total hospital’s consumption from pharmacy records as the number of vials for Imipenem, Meropenem, and Ertapenem, and converting them to defined daily doses (DDD) per 1000 patient days (DDD/1000PD) per quarter. The DDD, according to the Anatomical Therapeutic Chemical (ATC) /DDD system developed by The World Health Organization (WHO)’s Collaborating Centre for Drug Statistics Methodology, is a standardized measure of drug consumption that represents the assumed average maintenance dose per day for a drug used in its main indication in adults [9]. The total carbapenem consumption per quarter was then summed. The pharmacy records, maintained as part of audit control duties, ensured the completeness and accuracy of the dispensed antimicrobial data.

For ICU carbapenem consumption, no separate DDD/1000PD data were available. However, data on the days of therapy per 1,000 patient-days (DOT/1000PD) for carbapenem in ICU were available from the monthly infection control surveillance records and were subsequently aggregated on a quarterly and semester basis. This approach offers the advantage of providing a more precise measure of antibiotic use, which can improve the accuracy of monitoring and forecasting resistance patterns.

### Data analysis

All analyses were conducted using R software version 4.2.2 [10]. A significance level of 0.05 was used in all analyses.

Exploratory data analysis involved visualizing time series for carbapenem consumption and % resistance exhibited by *Klebsiella* spp. and determining trends using simple linear regression.

To examine potential association between carbapenem % resistance and consumption, a cross-correlation analysis (Spearman test) was employed using various time lags. All lags in carbapenem consumption showing significant correlation with % resistance were used in identifying the best forecasting model for % carbapenem resistance.

To determine the appropriate period for consumption to be used in training the forecasting models, trend and level changes in carbapenem consumption after Q1 2020 were examined for both total hospital’s DDD/1000PD and ICU’s DOT/1000PD to avoid potential historical bias arising from the effect of admission of COVID-19 cases starting from Q2 2020. Accordingly, the pre-COVID-19 period was defined as Q1 2019 to Q1 2020, while the post-COVID-19 period was defined as Q2 2020 to Q4 2023.

Classical time-series approaches were selected a priori for model development. Although alternative hybrid or machine-learning–based forecasting methods are increasingly used in antimicrobial resistance research, ARIMA and exponential smoothing models were chosen because of their transparency, suitability for relatively short time series, and ease of clinical interpretability. These characteristics were considered particularly important for stewardship-focused applications, where understanding temporal patterns and the contribution of lagged antibiotic consumption is essential for informing practice.

To identify the best forecasting models for carbapenem consumption in the hospital and ICU, a comprehensive evaluation was conducted on various Seasonal Autoregressive Integrated Moving Average (SARIMA) and exponential smoothing models. The best SARIMA model was identified using the ‘auto.arima’ function from the ‘forecast’ package in R [11]. The best exponential smoothing model was determined using the ‘ets’ function, considering Error, Trend (and damped Trend), and Seasonal decomposition (ETS). The Corrected Akaike Information Criterion (AICc) for each model was used as a model fit index while prediction accuracy was assessed using the root mean square error (RMSE) derived from time series cross-validation with a rolling forecasting origin. Under this approach, models were repeatedly refitted on expanding training windows and evaluated on subsequent observations. Forecast errors were calculated at each origin and averaged to obtain the RMSE, providing an estimate of out-of-sample predictive performance while preserving the temporal structure of the data. This approach is particularly appropriate for short time series, where conventional split-sample validation would substantially reduce the available training data and yield unstable estimates [12]. The two models with highest prediction accuracy (lowest RMSE) were identified and used to forecast Carbapenem consumption in total hospital and ICU up to Q4 2029 and S2 2029, respectively.

To identify the best forecasting models for the logit-transformed percentage of carbapenem resistance exhibited by *Klebsiella* spp. in the hospital and ICU, an evaluation was conducted on various SARIMA models with exogenous regressors (xreg) using sequential combinations of one to four lagged hospital carbapenem consumption and one to two lagged ICU carbapenem consumption. This analysis specifically accounted for the bounded nature of percentage data by using the logit transformation.

Lag selection for the final forecasting models was based on a combination of biological plausibility, parsimony, and predictive performance, with preference given to models achieving lower RMSE while minimizing overfitting given the limited number of time points.

Finally, forecasted carbapenem consumption in the hospital (DDD/1000 PD) and ICU (DOT/1000 PD) were used as regressors to forecast the logit-transformed percentage carbapenem resistance using the two best-identified models for the total hospital and ICU up to Q4 2029 and S2 2029, respectively. After forecasting, the predicted logit-transformed percentages were back-transformed to the original percentage scale to provide interpretable results.

Sensitivity analyses were conducted to evaluate the impact of alternative carbapenem consumption training strategies on resistance forecasts, including models trained using the full 2019–2023 consumption period and using the average pre-COVID-19 consumption level.

### Sample size

The study analyzed the complete records of % carbapenem resistance, encompassing a total of 1,727 *Klebsiella* spp. isolates collected from the hospital, including 1,043 from the ICU, over a four-year period from January 2020 to December 2023.

## Results

### Exploratory data analysis for trends in carbapenems’ % resistance and consumption

**Fig. 1:**
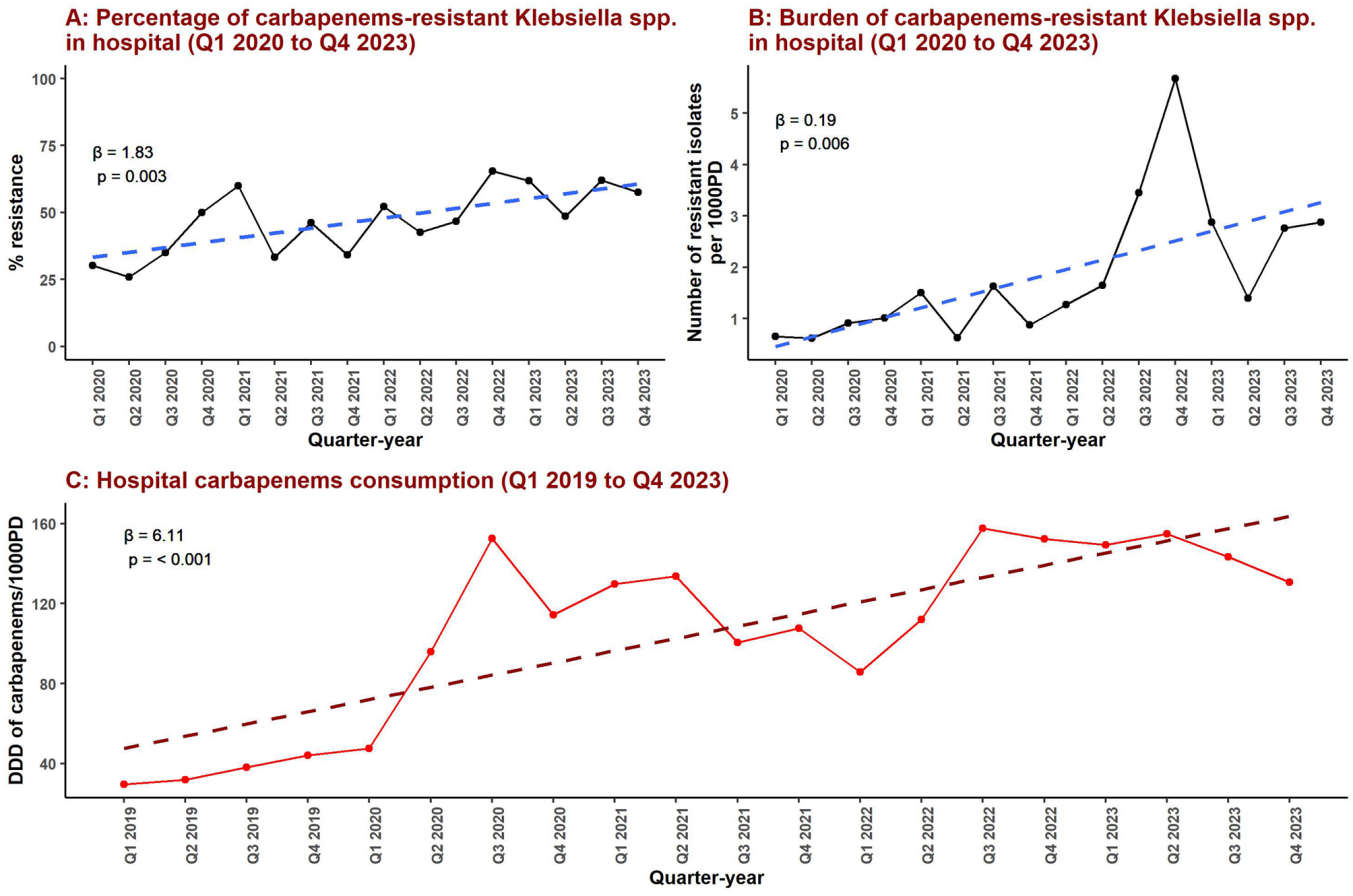
Temporal trends in carbapenem-resistant *Klebsiella* spp. prevalence, resistance burden, and carbapenem consumption in the total hospital. Panel **A** shows the quarterly percentage of carbapenem-resistant Klebsiella spp. among hospital isolates from Q1 2020 to Q4 2023. Panel **B** depicts the quarterly burden of carbapenem-resistant Klebsiella spp., expressed as the number of resistant isolates per 1,000 patient-days over the same period. Panel **C** illustrates quarterly total hospital carbapenem consumption from Q1 2019 to Q4 2023, expressed as defined daily doses per 1,000 patient-days (DDD/1,000 PD). Dashed lines represent fitted linear trends, with corresponding regression coefficients (β) and p-values shown within each panel

Figure 1; Table 1 summarize the temporal patterns of carbapenem resistance and carbapenem consumption in the total hospital. As shown in Fig. 1A and detailed in Table 1, the percentage of carbapenem-resistant *Klebsiella* spp. increased from 30.2% in Q1 2020 (95% CI: 20.6% to 39.8%) to 57.5% in Q4 2023 (95% CI: 49.3% to 65.7%), corresponding to a significant upward trend over time (β = 1.83, 95% CI: 0.75 to 2.91, *p* = 0.003). In parallel, Fig. 1B; Table 1 demonstrate a concomitant increase in the burden of carbapenem-resistant *Klebsiella* spp., expressed as the number of resistant isolates per 1,000 patient-days. Total hospital carbapenem consumption also rose substantially over the study period, increasing from 30 DDD/1,000 patient-days in Q1 2019 to 131 DDD/1,000 patient-days in Q4 2023 (Fig. 1C), with a significant positive temporal trend (β = 6.11, 95% CI: 3.86 to 8.35, *p* < 0.001). Table 1 also shows consistently high proportions of multidrug-resistant (MDR) Klebsiella spp. across the study period, which are reported to provide epidemiological context but were not examined analytically within the scope of the present study.

**Table 1:** Quarterly carbapenem consumption and carbapenem-resistant Klebsiella spp. burden and resistance rates in the total hospital, January 2019 to December 2023 (1727 Klebsiella spp. isolates)

Quarter	Patient days (PD)	Carbapenem consumption (DDD/1000 PD)	Total AMC (DDD/1000 PD)	Percentage of carbapenem consumption from total antibiotic consumption	Total bacterial isolates	Number of Klebsiella spp. isolates (% from Total bacterial isolates)	Number of carbapenem- resistant Klebsiella spp.		Percentage of carbapenem-resistant Klebsiella spp. (95% CI)	% MDR (95% CI)
							Absolute number	Per 1000 PD		
Q1 2019	33,138	30	645	4.6%						
Q2 2019	34,732	32	725	4.4%						
Q3 2019	36,562	38	655	5.8%						
Q4 2019	37,536	44	716	6.2%						
Q1 2020	38,086	48	766	6.2%	398	82 (20.6%)	25	0.65	30.2% (20.6% to 39.8%)	95% (90.5% to 99.8%)
Q2 2020	22,404	96	1183	8.1%	233	53 (22.7%)	14	0.61	25.9% (17.8% to 33.9%)	85% (75.3% to 94.5%)
Q3 2020	24,889	153	1224	12.5%	219	65 (29.7%)	23	0.91	35.0% (26.1% to 44.0%)	85% (75.8% to 93.4%)
Q4 2020	31,111	114	1116	10.3%	230	63 (27.4%)	31	1.01	50.0% (10.0% to 90.0%)	90% (83.2% to 97.7%)
Q1 2021	26,822	130	1241	10.5%	283	67 (23.7%)	40	1.50	60.0% (26.1% to 93.9%)	90% (82.2% to 96.9%)
Q2 2021	29,256	134	1234	10.8%	232	55 (23.7%)	18	0.63	33.3% (0.0% to 86.6%)	84% (73.9% to 93.4%)
Q3 2021	28,673	100	986	10.2%	370	101 (27.3%)	47	1.63	46.2% (37.7% to 54.7%)	90% (84.3% to 95.9%)
Q4 2021	33,656	108	996	10.8%	354	86 (24.3%)	29	0.87	34.1% (24.2% to 44.1%)	91% (84.6% to 96.8%)
Q1 2022	33,010	86	923	9.3%	363	80 (22.0%)	42	1.27	52.2% (42.1% to 62.4%)	90% (83.4% to 96.6%)
Q2 2022	31,989	112	964	11.6%	405	124 (30.6%)	53	1.65	42.6% (35.6% to 49.5%)	87% (81.2% to 93.0%)
Q3 2022	24,548	158	1221	12.9%	565	181 (32.0%)	85	3.45	46.7% (40.3% to 53.2%)	95% (91.9% to 98.2%)
Q4 2022	27,204	152	1117	13.6%	661	236 (35.7%)	154	5.67	65.4% (59.8% to 71.0%)	93% (89.5% to 96.1%)
Q1 2023	29,643	149	1060	14.1%	485	138 (28.5%)	85	2.88	61.8% (54.7% to 68.9%)	89% (83.9% to 94.3%)
Q2 2023	30,556	155	1043	14.9%	382	88 (23.0%)	43	1.40	48.6% (40.5% to 56.6%)	89% (82.0% to 95.3%)
Q3 2023	31,882	143	1033	13.9%	486	142 (29.2%)	88	2.76	62.0% (51.3% to 72.7%)	77% (70.6% to 84.3%)
Q4 2023	33,150	131	1023	12.8%	468	166 (35.5%)	95	2.88	57.5% (49.3% to 65.7%)	72% (65.5% to 79.1%)

PD, patient-days; DDD, defined daily dose; AMC, antimicrobial consumption; MDR, multidrug-resistant. Carbapenem consumption and total AMC are expressed as DDD per 1,000 patient-days. Carbapenem resistance percentages and MDR proportions are presented with 95% confidence intervals, calculated using binomial methods. Absolute resistance burden is additionally expressed per 1,000 patient-days to account for variations in hospital activity over time. Quarters with fewer than 30 Klebsiella spp. isolates were retained for descriptive purposes but interpreted with caution. Resistance data were available from Q1 2020 onward, whereas carbapenem consumption data were available from Q1 2019 and included to assess their temporal effects on subsequent resistance patterns

**Fig. 2:**
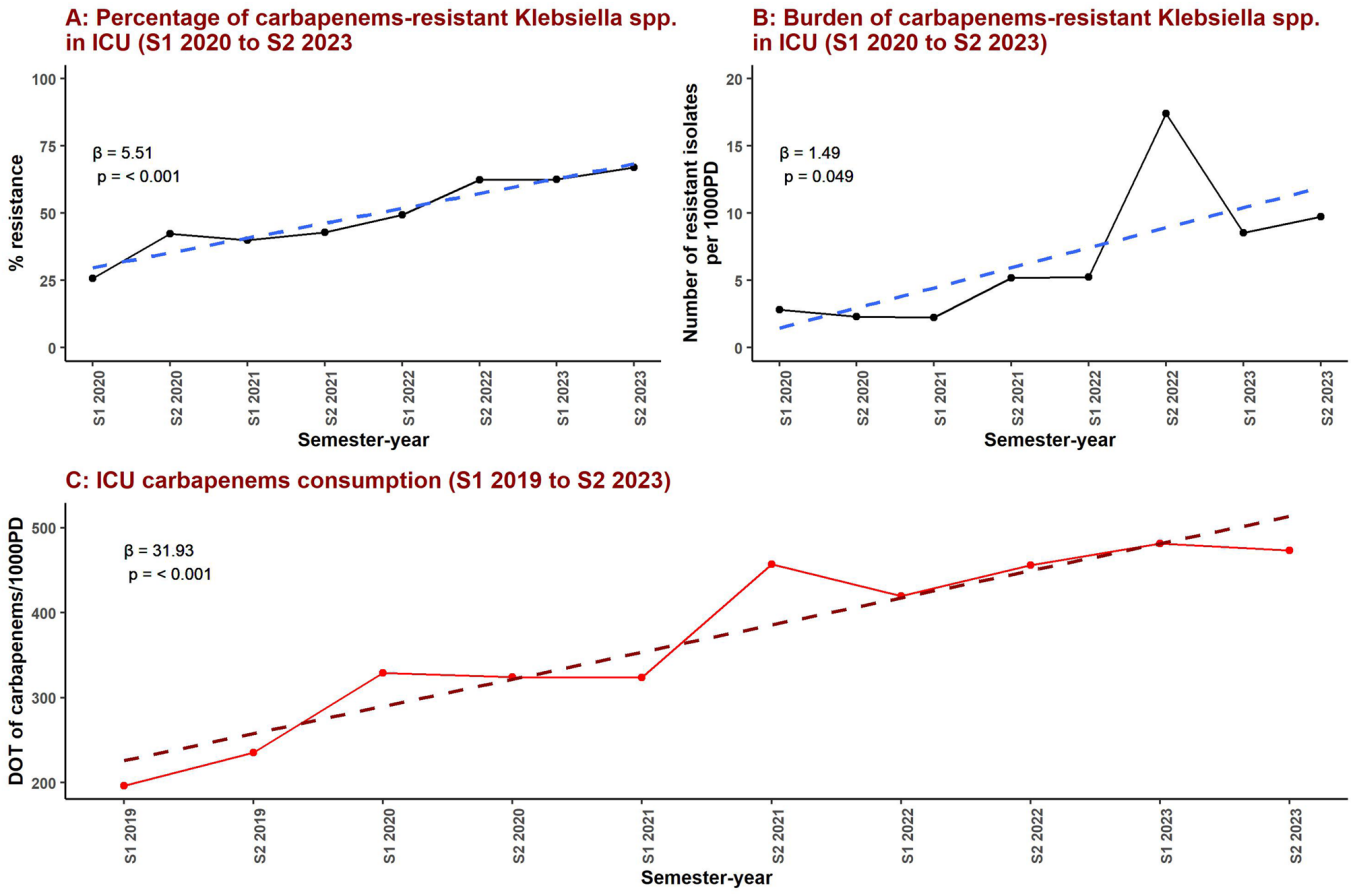
Temporal trends in carbapenem-resistant Klebsiella spp. prevalence, resistance burden, and carbapenem consumption in the intensive care unit. Panel **A** shows the semester-level percentage of carbapenem-resistant Klebsiella spp. among ICU isolates from S1 2020 to S2 2023. Panel **B** depicts the semester-level burden of carbapenem-resistant Klebsiella spp., expressed as the number of resistant isolates per 1,000 patient-days over the same period. Panel **C** illustrates ICU carbapenem consumption from S1 2019 to S2 2023, expressed as days of therapy per 1,000 patient-days (DOT/1,000 PD). Dashed lines represent fitted linear trends, with corresponding regression coefficients (β) and p-values shown within each panel

Figure 2; Table 2 summarize the temporal patterns of carbapenem resistance and carbapenem consumption within the ICU. As shown in Fig. 2A and detailed in Table 2, the percentage of carbapenem-resistant *Klebsiella* spp. increased from 25.8% in S1 2020 (95% CI: 18.5% to 33.1%) to 67.0% in S2 2023 (95% CI: 58.6% to 75.3%), corresponding to a significant upward trend over time (β = 5.51, 95% CI: 3.89 to 7.13, *p* < 0.001). In parallel, Fig. 2B; Table 2 demonstrate a marked increase in the burden of carbapenem-resistant Klebsiella spp., expressed as the number of resistant isolates per 1,000 patient-days. ICU carbapenem consumption also rose substantially over the study period, increasing from 196 DOT/1,000 patient-days in S1 2019 to 473 DOT/1,000 patient-days in S2 2023 (Fig. 2C), with a significant positive temporal trend (β = 31.93, 95% CI: 22.70 to 41.17, *p* < 0.001).

**Table 2:** Semester-level carbapenem consumption and carbapenem-resistant Klebsiella spp. burden and resistance rates in the intensive care unit, January 2019 to December 2023 (1043 Klebsiella spp. isolates)

Semester	Patient days (PD)	carbapenem consumption (DOT/1000 PD)	Total AMC (DOT/1000 PD)	Percentage of carbapenem consumption from total antibiotic consumption	Total bacterial isolates	Number of Klebsiella spp. isolates (% from Total bacterial isolates)	Number of carbapenems- resistant Klebsiella spp.		Percentage of carbapenem-resistant Klebsiella spp. (95% CI)	% MDR (95% CI)
							Absolute number	Per 1000 PD		
S1 2019	6485	196	649	30.3%						
S2 2019	9020	235	717	32.8%						
S1 2020	8778	329	769	42.8%	356	96 (27.0%)	25	2.82	25.8% (18.5% to 33.1%)	92.7% (87.5% to 97.9%)
S2 2020	10,305	324	750	43.2%	167	56 (33.5%)	24	2.30	42.3% (31.6% to 53.0%)	91.1% (83.6% to 98.5%)
S1 2021	9606	324	857	37.8%	193	54 (28.0%)	22	2.25	40.0% (40.0% to 40.0%)	90.7% (83.0% to 98.5%)
S2 2021	10,233	457	914	50.0%	397	124 (31.2%)	53	5.20	42.9% (34.9% to 50.9%)	91.9% (87.1% to 96.7%)
S1 2022	10,906	419	872	48.1%	398	116 (29.1%)	57	5.25	49.4% (41.7% to 57.1%)	91.4% (86.3% to 96.5%)
S2 2022	9934	456	900	50.7%	708	277 (39.1%)	173	17.39	62.4% (57.3% to 67.5%)	94.9% (92.4% to 97.5%)
S1 2023	10,376	482	842	57.2%	444	142 (32.0)	89	8.55	62.5% (55.9% to 69.2%)	93.0% (88.7% to 97.2%)
S2 2023	12,221	473	804	58.8%	460	178 (38.7)	119	9.75	67.0% (58.6% to 75.3%)	82.6% (77.0% to 88.2%)

PD, patient-days; DOT, days of therapy; AMC, antimicrobial consumption; MDR, multidrug-resistant. Carbapenem consumption and total AMC are expressed as DOT per 1,000 patient-days. Carbapenem resistance percentages and MDR proportions are presented with 95% confidence intervals, calculated using binomial methods. Absolute resistance burden is additionally expressed per 1,000 patient-days to contextualize resistance patterns relative to ICU activity. ICU resistance data were aggregated at the semester level to ensure reliable estimates in accordance with CLSI recommendations for antibiogram reporting. Resistance data were available from S1 2020 onward, whereas carbapenem consumption data were available from S1 2019 and included to assess their temporal effects on subsequent resistance patterns

### Correlations between carbapenems’ % resistance and consumption

Table 3 summarizes the correlations between the % carbapenem resistance in *Klebsiella* spp. and carbapenem consumption in total hospital (DDD/1000PD) and ICU settings (DOT/1000PD) with different time lags. For hospital’s % carbapenem resistance per quarter, significant correlations were observed with both the previous quarter (*r* = 0.80, *p* < 0.001) and the penultimate quarter (*r* = 0.60, *p* = 0.015). In the ICU setting, the correlations between % carbapenem resistance per semester and each of the previous semester and penultimate semester consumptions were significant with *r* = 0.86 (*p* = 0.011) and *r* = 0.76 (*p* = 0.037), respectively.

**Table 3:** Correlations between percentage of carbapenem resistance exhibited by Klebsiella spp. and carbapenem consumption in total hospital and ICU within different time lags in a Tertiary Hospital in Alexandria, Egypt from 2019 to 2023

Hospital’s % carbapenem resistance per quarter			ICU’s % carbapenem resistance per semester		
Quarter Consumption lags	*r*	*p*	Semester Consumption lags	*r*	*p*
Lag 1	0.80	< 0.001*	Lag 1	0.86	0.011*
Lag 2	0.60	0.015*	Lag 2	0.76	0.037*
Lag 3	0.28	0.299			
Lag 4	0.41	0.12			

r: Spearman correlation coefficient. For hospital consumption, Lag1 refers to the carbapenem consumption in DDD/1000PD from the previous quarter, Lag2 from two quarters prior, Lag3 from three quarters earlier, and Lag4 indicates the value from four quarters ago. For ICU consumption, Lag1 refers to the carbapenem consumption in DOT/1000PD from the previous semester, Lag2 from two semesters prior. *Statistically significant (*p* < 0.05)

### Changes in carbapenem consumption during the COVID-19 pandemic

**Fig. 3:**
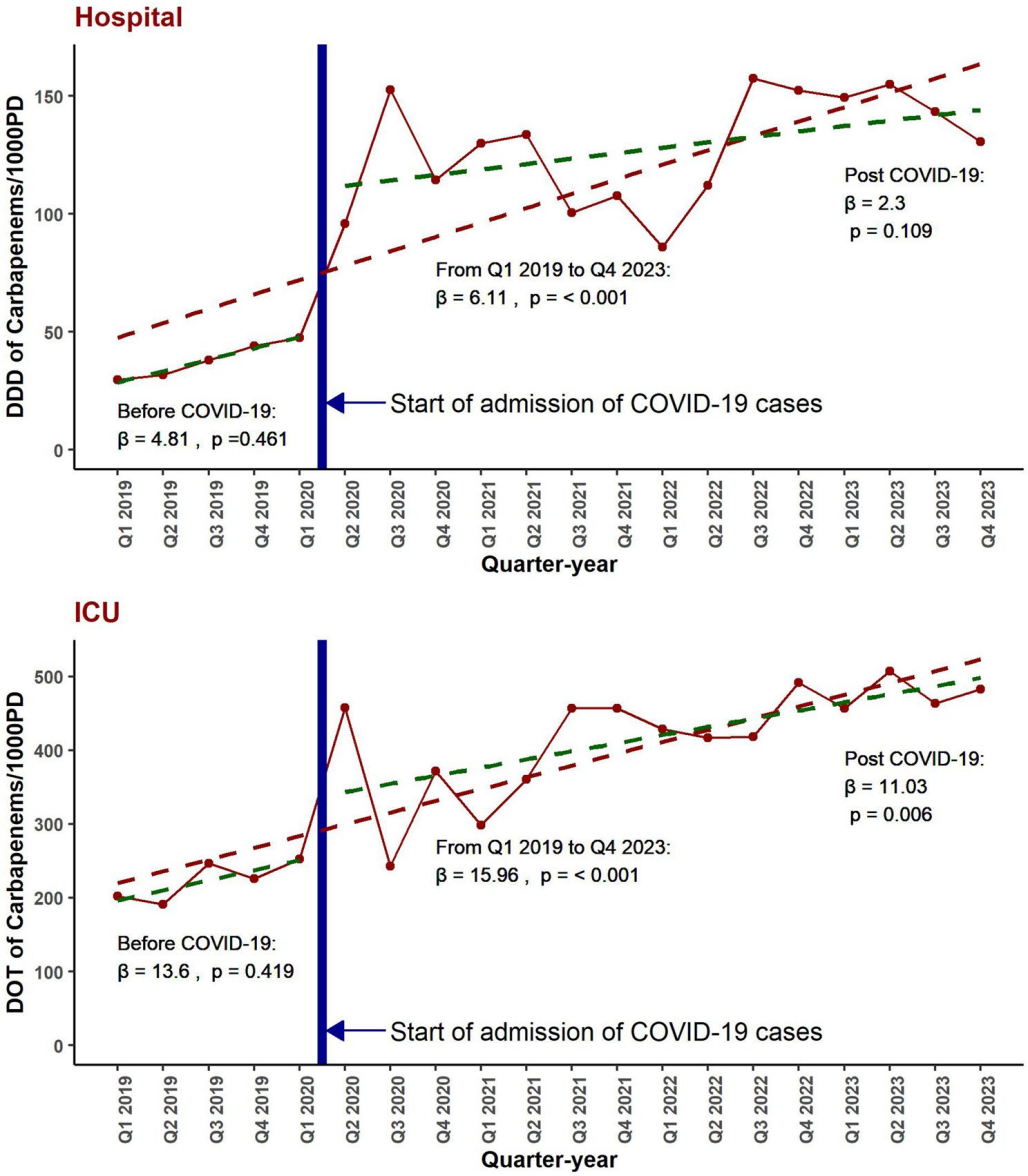
Impact of COVID-19 on carbapenem consumption trends. Top panel: Total hospital carbapenem consumption (DDD/1000PD) increased significantly overall, with a pronounced increase in the level post-pandemic. Bottom panel: ICU carbapenem consumption (DOT/1000PD) also increased significantly, with a more notable rise post-pandemic. DDD/1000PD: Defined daily doses per 1000 patient days. DOT/1000PD: Days of therapy per 1000 patient days. β: regression coefficient

Figure 3 provides an expanded overview of carbapenem consumption trends in the total hospital and ICU from January 2019 to December 2023, highlighting the impact of the COVID-19 pandemic by dividing the data into pre- and post-pandemic periods. The top panel shows that total hospital carbapenem consumption, measured in DDD/1000PD, increased significantly over the entire study period (β = 6.11, *p* < 0.001). However, this increase was not consistent across both segments, with no significant rise pre-pandemic (β = 4.81, *p* = 0.461) or post-pandemic (β = 2.3, *p* = 0.109). Similarly, the bottom panel and Supplementary Table S1 displays ICU carbapenem consumption in DOT/1000PD, which also increased significantly over the entire period (β = 15.96, *p* < 0.001). While the pre-pandemic trend was not statistically significant (β = 13.6, *p* = 0.419), the post-pandemic increase was significant (β = 11.03, *p* = 0.006).

Table 4 further supports these findings, highlighting the impact of the COVID-19 pandemic on consumption levels and trends. Although the level of hospital carbapenem consumption increased significantly post-pandemic onset (β = 61.82, *p* = 0.005*), the level change in ICU consumption was not statistically significant (*p* = 0.116). Trend changes for both settings were not significant, indicating that the rate of increase in carbapenem consumption did not differ significantly post-pandemic.

**Table 4:** Impact of the COVID-19 Pandemic on Level and Quarterly Trend Changes of Carbapenem Consumption in Total hospital and ICU in a Tertiary Hospital in Alexandria, Egypt from 2019 to 2023

Term	Hospital Carbapenem consumption (DDD/1000PD)	ICU Carbapenem consumption (DOT/1000PD)
	β (95% CI)	β (95% CI)
(Intercept)	23.79 (-20.98, 68.57)	182.93 (67.59, 298.28)*
Quarterly trend before COVID-19	4.81 (-8.69, 18.31)	13.60 (-21.18, 48.38)
Level change after COVID-19	61.82 (21.42, 102.21)*	81.48 (-22.58, 185.53)
Quarterly trend change after COVID-19	-2.51 (-16.25, 11.23)	-2.57 (-37.96, 32.83)

DDD/1000PD: Defined daily dose per 1000 patient days. DOT/1000PD: Days of therapy per 1000 patient days. β: regression coefficient representing change in DDD/1000PD or DOT/1000PD. CI: confidence interval. *Statistically significant (*P* < 0.05)

### Forecasting percentage of carbapenem resistance

A summary of the model evaluation process is available in Supplemental Tables S2-S3 and Supplementary Figures S1-S4 presenting the model fit and prediction accuracy indices for the best forecasting SARIMA and ETS models for predicting carbapenem consumption and the best SARIMA models for forecasting the percentage of carbapenem resistance using various time lags. The models with the highest prediction accuracy (least RMSE) were as follows (highlighted in grey in Supplementary Table S2): For hospital carbapenem consumption, the ARIMA (0,0,0) model, trained on data from Q2 2020 to 2023, demonstrated an RMSE of 23.42. For ICU carbapenem consumption, the ETS (MAN, damped = True) model, trained on data from S1 2019 to S2 2023, showed the highest prediction accuracy with an RMSE of 46.65. For forecasting the logit-transformed percentage of carbapenem resistance in hospitals, the ARIMA (0,1,1) model with exogenous regressors (lag1 quarter consumption) had the best prediction accuracy with an RMSE of 0.40. For ICU models forecasting the logit-transformed percentage of carbapenem resistance, the ARIMA (0,1,0) with exogenous regressors (lag 1 semester consumption) achieved the highest accuracy with an RMSE of 0.43.

**Fig. 4:**
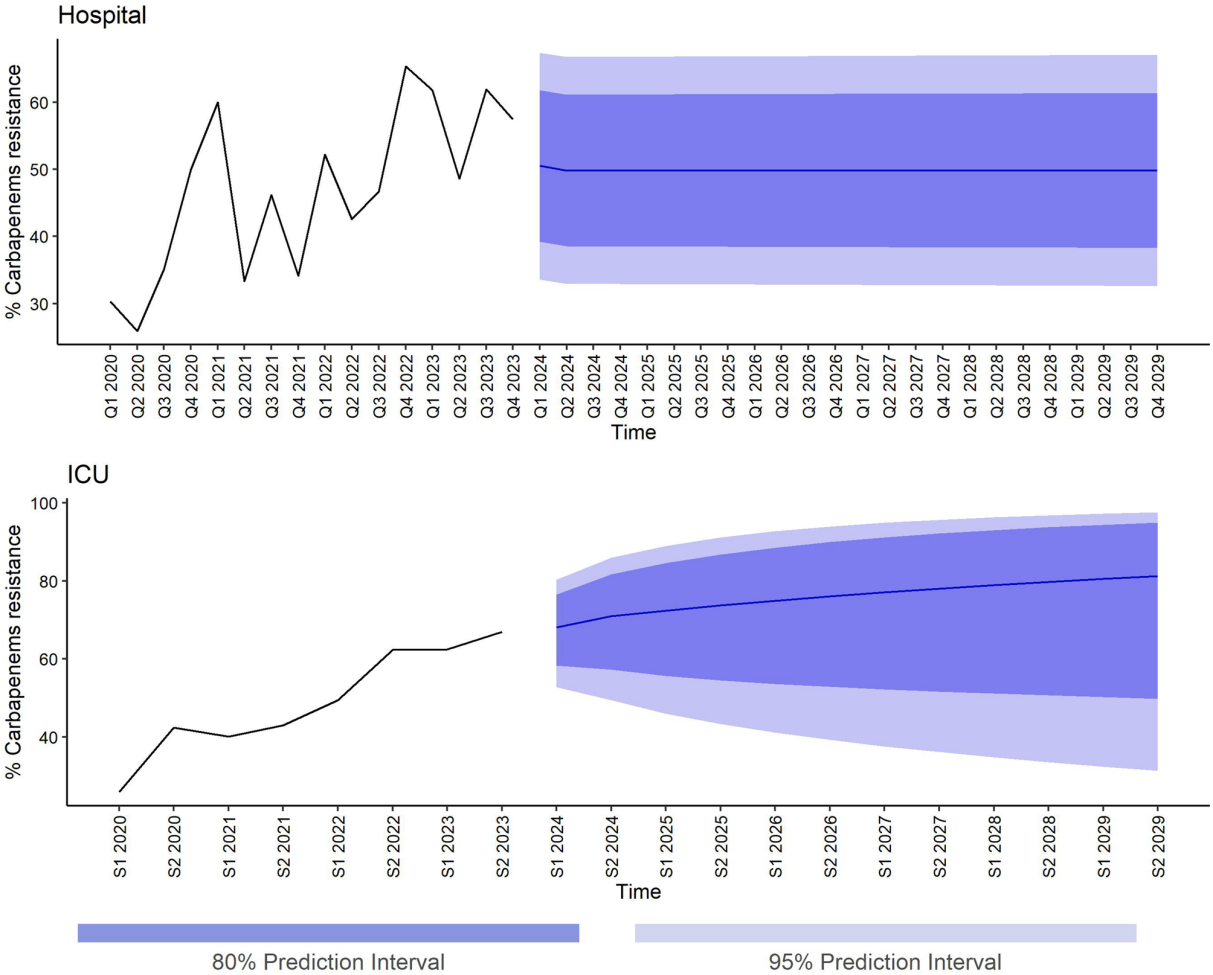
Forecasted trends in carbapenem resistance in *Klebsiella* spp. Top panel: Predicted stationary carbapenem resistance in the hospital based on post-COVID-19 consumption data. Bottom panel: Forecasted steady increase in carbapenem resistance within the ICU, with a potential for near-complete resistance by 2029

Figure 4 presents forecasted trends in carbapenem resistance exhibited by *Klebsiella* spp. based on projected carbapenem consumption within the hospital (top panel) and ICU (bottom panel).

The top panel depicts forecasts of carbapenem resistance based on a DDD/1000PD ARIMA (0,1,1) model trained on post-COVID-19 consumption data (Q2 2020 to Q4 2023). The model predicts stationary carbapenem resistance with a mean of 49.8% and a relatively wide 95% prediction interval (PI) (32.6% to 67.1%). The bottom panel illustrates forecasts of carbapenem resistance based on a DOT/1000PD ETS(M, A_d_,N) model trained on consumption data from S1 2019 to S2 2023. The model predicts a steady increase in carbapenem resistance within the ICU. The expected carbapenem resistance at the end of 2029 is 81.2% (95% PI: 31.3% to 97.6%). These hospital- and ICU-level forecasts should be interpreted within their respective settings, as carbapenem consumption was quantified using different metrics (DDD/1000PD for the hospital and DOT/1000PD for the ICU), which capture distinct aspects of antibiotic exposure and do not permit direct quantitative comparison across settings.

To provide a complete presentation of forecast uncertainty, all point forecasts with corresponding 80% and 95% prediction intervals for both hospital and ICU models are presented in Supplementary Table S4. Across the hospital forecasts, prediction intervals remained relatively stable over time, with only minimal widening at longer horizons (e.g., 49.8% in Q4 2026; 95% PI: 32.8% to 66.9%, compared with Q4 2029; 95% PI: 32.6% to 67.1%), a difference that is not visually appreciable in Fig. 4. In contrast, ICU forecasts exhibited wider prediction intervals overall, which were already substantial at intermediate horizons (e.g., 76.0% in S2 2026; 95% PI: 39.2% to 94.0%) and expanded further toward the end of the projection period (81.2% in S2 2029; 95% PI: 31.3% to 97.6%), a pattern that is visually evident in Fig. 4.

Sensitivity analyses were conducted to assess the impact of alternative hospital carbapenem consumption training strategies on resistance forecasts. When the consumption model was trained using the full 2019–2023 dataset, forecasted carbapenem resistance stabilized at a mean of 50.6%, with prediction intervals largely overlapping those of the primary analysis (95% PI: 33.3% to 67.7% by 2029; Supplementary Table S5 and Figure S5A). In contrast, models trained using the average pre-COVID-19 carbapenem consumption level (38.4 DDD/1000PD) yielded substantially lower projected resistance, with a stable mean of 26.7% and narrower uncertainty bounds (95% PI: 15.0% to 42.7% by 2029; Supplementary Table S5 and Figure S5B).

## Discussion

The current study reveals a concerning upward trajectory in carbapenem resistance among *Klebsiella* spp. both in the hospital and ICU settings, paralleled by a substantial increase in carbapenem consumption. A marked and sustained increase in hospital carbapenem consumption was observed following the onset of the COVID-19 pandemic, occurring in temporal association with the pandemic period.

The significant increase in carbapenem resistance exhibited by *Klebsiella* spp. post-pandemic in Egypt was also observed in a recent study by Shaimaa Abdelaziz Abdelmoneim et al. (2024) that mapped AMR pre- and post-COVID-19 pandemic and proved that overuse of antibiotics during coronavirus disease 2019 (COVID-19) in an attempt to reduce COVID-19 mortality in the short term resulted in an increased *Klebsiella pneumoniae* resistance against quinolones and carbapenem [12]. These trends underscore the urgent need for effective antimicrobial stewardship programs to mitigate the emergence and dissemination of carbapenem-resistant organisms in Egypt.

The strong positive correlations observed between carbapenem resistance and consumption at shorter time lags indicate a strong temporal association between carbapenem consumption and resistance, with shorter lags suggesting a rapid emergence of resistance following increased antibiotic exposure. This aligns with previous research that used cross-correlation functions to identify significant time lags between carbapenem consumption and resistance time series, finding that increased carbapenem use is a major driver of resistance development [13, 14]. However, these findings should be interpreted as associative rather than causal, as potential confounders—including infection control policy changes, staffing changes, formulary modifications, and patient comorbidities—could not be formally controlled.

The modeling results in the present study indicate that relatively simple models effectively predicted carbapenem consumption and resistance trends. While the ARIMA(0,0,0) model accurately forecast hospital carbapenem consumption, the ETS(MAdN) model excelled in predicting ICU consumption. Notably, incorporating only one lagged carbapenem consumption as an exogenous variable in ARIMA models in both total hospital and ICU settings showed best resistance prediction accuracy. The hospital demonstrated forecasted stable level of carbapenem resistance, likely due to the stabilization of post-COVID-19 hospital carbapenem consumption after the initial significant increase, however there is a concerningly wide prediction interval for carbapenem resistance, suggesting potential variability in future trends. Conversely, the ICU model exhibited a steadier increase in resistance, likely due to the continuing positive trend in ICU carbapenem consumption in DOT/1000PD.

The use of different training periods for hospital and ICU consumption models reflects differences in both underlying consumption dynamics and the metrics used to quantify antibiotic exposure. At the hospital level, carbapenem consumption measured as DDD/1000 patient-days demonstrated a clear and statistically significant level change following the onset of the COVID-19 pandemic, indicating a shift to a new consumption regime. In contrast, ICU carbapenem consumption, measured using DOT/1000 patient-days, exhibited a more gradual and continuous upward trend over time without an abrupt level shift. These differences justified restricting hospital-level model training to the post-COVID-19 period, while allowing ICU models to incorporate the full available time series starting from S1 2019 to capture longer-term trends.

Importantly, trends observed at the hospital and ICU levels should not be interpreted as directly comparable in magnitude or severity. Hospital carbapenem consumption was quantified using DDD/1000 patient-days, a standardized population-level metric, whereas ICU consumption was measured using DOT/1000 patient-days, which reflects patient-level antibiotic exposure. Consequently, differences in forecasted resistance trajectories reflect setting-specific dynamics rather than relative burden across settings.

Sensitivity analyses further support the robustness of the chosen modeling strategy for hospital-level forecasts. Resistance projections derived from models trained on the full 2019–2023 consumption period were largely comparable to those of the primary post-COVID-19 analysis, with overlapping prediction intervals and only marginally higher forecasted resistance levels. In contrast, the model based on average pre-COVID-19 consumption levels yielded substantially lower long-term resistance estimates, reflecting extrapolation from a consumption regime that no longer characterizes contemporary hospital practice. Together, these findings indicate that restricting model training to the post-COVID-19 period did not materially alter resistance forecasts compared with using the full dataset, while providing projections that are more consistent with current prescribing patterns and stewardship-relevant decision-making.

The study demonstrated several strengths. Firstly, this study leveraged both the practical aggregate-level hospital data (DDD/1000PD) and the guidelines-preferred granular patient-level ICU surveillance data (DOT/1000PD), providing complementary perspectives on carbapenem utilization and resistance patterns within distinct clinical contexts.

Secondly, this study comprehensively examined the cross-correlation between carbapenem resistance focusing on *Klebsiella* spp. (the top isolated bacterial pathogen in Egyptian hospitals and ICUs) and consumption by incorporating various time lags, providing valuable insights into the dynamic relationship between these variables. X Wu et al. study examined the correlations between lagged consumption and resistance using only Meropenem to represent the carbapenem, the study found correlation between Meropenem consumption and Meropenem resistant K. pneumoniae peaking at 0-quarter lag [13]. On the other hand Y Hao et al. study only examined the correlation between carbapenem consumption and Imipenem-resistant *Klebsiella pneumoniae* but found no significant correlations [14].

Thirdly, this study contributes to the limited body of research employing dynamic regression models, as highlighted by a recent systematic review by Paul Laffon-Lozes et al. (2023), which screened 641 articles and identified only 28 studies using this methodology to assess the impact of antimicrobial consumption on AMR [15]. Of these, only three studies examined the relationship between carbapenem use and carbapenem-resistant Klebsiella spp., using consumption data lagged by 0 quarters, 1 quarter, six months, and one year as predictors of resistance [16–18]. The present study effectively proved the significance of incorporating lagged carbapenem consumption as exogenous regressors in dynamic regression models to enhance the prediction accuracy of forecasting carbapenem resistance using ARIMA models compared to models without such variables.

ARIMA models have proven beneficial for forecasting carbapenem-resistant *Klebsiella pneumoniae* and carbapenem consumption, as demonstrated in research by Xiaoqin Wu et al., who applied them to evaluate the effectiveness of digital-based transparent supervision in preventing and controlling carbapenem-Resistant Klebsiella pneumoniae nosocomial infections [19]. The study involved a substantial number of patient cultures, with 46,873 pre-intervention and 45,217 post-intervention. However, it did not investigate the effect of lagged consumption data as exogenous regressors.

This study has certain limitations, and the first limitation pertains to the ICU forecasting model for carbapenem resistance, which relied on a relatively small dataset encompassing only eight observations (semesters from 2020 to 2023). This was due to the aggregation of ICU resistance data into semesters to avoid potential bias arising from unreliable estimates resulting from a number of ICU isolates lower than the CLSI-recommended number to be included in antibiograms (less than 30) in many quarters [20]. Accordingly, the relatively wide 95% prediction intervals observed for the ICU forecasts should be interpreted as a realistic quantification of uncertainty driven by limited longitudinal data and inherent variability, rather than as a weakness of the forecasting model. Under this aggregation strategy, each ICU semester included at least 54 *Klebsiella* spp. isolates (1,043 isolates across eight semesters; Table 2), ensuring reliable antibiogram estimates in accordance with CLSI recommendations, while necessarily reducing temporal granularity and statistical power compared with quarter-level analyses [21]. Second, ICU carbapenem consumption data were derived from official infection control surveillance records rather than comprehensive ICU-wide administration databases; as such, estimates should be interpreted as reflecting temporal trends within the surveilled population rather than absolute ICU consumption levels. Third, despite that DDDs provide a practical solution for many hospitals and it is the WHO recommended strategy that ensures ongoing monitoring and stewardship as it may be calculated even in the absence of electronic pharmacy records by using purchasing data, DOT/1000PD is currently the most accurate and preferred measure of antibiotic use by the infectious disease society of America (IDSA) and is used by CDC and US National Healthcare Safety Network as it allows for multiple patient populations to be compared accurately and is not affected by changes in dosing or WHO DDD over time nor formulary differences between institutions [22, 23]. Accordingly, resistance trends and forecasts derived from DDD/1000PD and DOT/1000PD should be interpreted within their respective settings, and direct quantitative comparisons between hospital and ICU trajectories were intentionally avoided. Fourth, no formal documentation was available regarding changes in microbiological sampling, culturing, or infection control practices at the hospital or ICU level over the study period; therefore, while no such changes were reported institutionally, their potential influence on observed resistance patterns cannot be fully excluded.

Future local research should focus on digitalizing hospital pharmacy records as it is crucial to ensure the continuous and accurate monitoring of antibiotic usage and to explore the impact of different antimicrobial stewardship interventions on carbapenem consumption and resistance trends. Additionally, implementing digital health records would enhance monitoring capabilities, particularly through the use of the DOT/1000PD metric for all patients in the ICU as well as the total hospital. At present, hospital-level antibiotic consumption was available only as dispensed quantities, which were converted to DDD/1000 patient-days, while DOT-based surveillance was conducted exclusively in the ICU. The absence of hospital-level DOT data precluded direct cross-setting comparisons using a harmonized metric; future implementation of integrated electronic prescribing and medication administration systems may enable standardized DOT-based surveillance across care settings. However, it is crucial to recognize that most published consumption data globally rely on DDD/1000PD rather than DOT/1000PD, which presents a widespread challenge for consistency and comparison across studies.

The observed trends have profound implications for patient care and public health. The rising prevalence of carbapenem-resistant infections limits treatment options, increasing morbidity and mortality rates. Moreover, the financial burden associated with the management of these infections is substantial. Our findings emphasize the critical role of antimicrobial stewardship programs in optimizing antibiotic use and preventing the emergence of resistance. Targeted interventions aimed at reducing carbapenem consumption, particularly in high-risk settings like ICUs, are essential.

## Conclusions

This study illustrates the application of dynamic regression forecasting to antimicrobial resistance surveillance data. Persistently high carbapenem resistance at the hospital level and a continued upward trend within the ICU setting, each reflecting context-specific consumption dynamics. Given the limited number of ICU observations and the resulting uncertainty in long-term projections, forecasts should be interpreted cautiously and primarily as indicators of potential future risk rather than precise predictions. Nevertheless, these findings reinforce the need for sustained surveillance and support antimicrobial stewardship efforts aimed at monitoring trends and mitigating further resistance escalation in healthcare settings in Egypt.

## Supplementary Information

Below is the link to the electronic supplementary material.

Supplementary Material 1

## Data Availability

The datasets used and/or analyzed during the current study are available from the corresponding author on reasonable request.

## Abbreviations

AMR: antimicrobial resistance
AMS: antimicrobial stewardship
CLSI: Clinical and Laboratory Standards Institute
COVID-19: Coronavirus disease 2019
CRO: Carbapenem-resistant organisms
DDD/1000PD: defined daily doses per 1000 patient days
DOT/1000PD: Days of therapy per 1000 patient days
ICU: Intensive care unit
PI: prediction interval
WHO: World Health Organization
