# Supplementary material for: Dynamic regression forecasting of carbapenem-resistant Klebsiella spp. based on carbapenem consumption with observed data from 2020 to 2023 and projections up to 2029 in a tertiary hospital in Alexandria, Egypt
Source: BMC Infect Dis. 2026 Apr 11;26:767. doi: 10.1186/s12879-026-13168-y (PMC13085628; doi:10.1186/s12879-026-13168-y)
Supplement: Supplementary file 1 — Supplementary Material 1 [file 12879_2026_13168_MOESM1_ESM.docx]

**Supplementary materials**

| **Supplementary Table S1: Quarterly ICU carbapenem consumption and total antimicrobial use from January 2019 to December 2023 (DOT/1000 patient-days)** | | | |
| --- | --- | --- | --- |
| Quarter | Carbapenems Consumption (DOT/1000PD) | Total AMC (DOT/1000PD) | % Carbapenems from total consumption |
| Q1 2019 | 202 | 664 | 30.4% |
| Q2 2019 | 191 | 635 | 30.1% |
| Q3 2019 | 247 | 721 | 34.2% |
| Q4 2019 | 226 | 715 | 31.6% |
| Q1 2020 | 253 | 721 | 35.1% |
| Q2 2020 | 457 | 850 | 53.8% |
| Q3 2020 | 243 | 648 | 37.5% |
| Q4 2020 | 372 | 810 | 45.8% |
| Q1 2021 | 299 | 876 | 34.1% |
| Q2 2021 | 361 | 828 | 43.6% |
| Q3 2021 | 457 | 914 | 50.0% |
| Q4 2021 | 457 | 914 | 50.0% |
| Q1 2022 | 429 | 854 | 50.2% |
| Q2 2022 | 417 | 877 | 47.6% |
| Q3 2022 | 419 | 889 | 47.1% |
| Q4 2022 | 491 | 910 | 54.0% |
| Q1 2023 | 457 | 866 | 52.7% |
| Q2 2023 | 507 | 817 | 62.1% |
| Q3 2023 | 464 | 797 | 58.2% |
| Q4 2023 | 483 | 812 | 59.4% |

ICU carbapenem consumption is expressed as days of therapy per 1,000 patient-days (DOT/1000PD). Total antimicrobial consumption (AMC) represents the aggregate DOT/1000PD for all systemic antibiotics administered in the ICU. Quarterly data are presented to illustrate underlying temporal variability and were subsequently aggregated to the semester level in the main analyses to ensure stable estimates and compliance with CLSI recommendations for resistance surveillance.

| **Supplementary Table S2: Performance of the best forecasting autoregressive integrated moving average and exponential smoothing models for predicting Carbapenems consumption and best autoregressive integrated moving average models for forecasting logit-transformed % Carbapenems resistant *Klebsiella* spp. using various time lags** | | |
| --- | --- | --- |
| Model | Model fit (AICc) | Prediction accuracy (RMSE) |
| **Carbapenems consumption** |  |  |
| **Hospital models** |  |  |
| ARIMA (0,0,0) model for consumption trained on data from Q2 2020 to Q4 2023 | 141.69 | 23.42 |
| ETS (ANN) model for consumption trained on data from Q2 2020 to Q4 2023 | 143.44 | 27.7 |
| ARIMA (0,1,0) model for consumption trained on data from Q1 2019 to Q4 2023 | 178.59 | 28.29 |
| ETS (MNN) model for consumption trained on data from Q1 2019 to Q4 2023 | 198.79 | 25.27 |
| **ICU models** |  |  |
| ARIMA (0,1,0) model for consumption trained on data from S1 2019 to S2 2023 | 101.58 | 61.32 |
| ARIMA (0,1,0) model for consumption trained on data from S1 2020 to S2 2023 | 78.80 | 59.52 |
| ARIMA (0,1,0) model for consumption trained on data from S2 2020 to S2 2023 | 69.06 | 65.20 |
| ETS (MAN, damped = True) model for consumption trained on data from S1 2019 to S2 2023 | 133.16 | 46.65 |
| ETS (MNN) model for consumption trained on data from S1 2020 to S2 2023 | 93.35 | 63.92 |
| ETS (ANN) model for consumption trained on data from S2 2020 to S2 2023 | 83.36 | 69.88 |
| **% Carbapenem-resistant *Klebsiella* spp.** |  |  |
| **Hospital models** |  |  |
| ARIMA (0,0,0) with exogenous regressors (lag1 quarter consumption) | 16.2 | 0.48 |
| ARIMA (0,1,1) with exogenous regressors (lag1 quarter consumption) | 19.7 | 0.40 |
| ARIMA (0,0,0) with exogenous regressors (lag1+ lag2 quarters’ consumptions) | 18.6 | 0.61 |
| ARIMA (0,0,0) with exogenous regressors (lag1+ lag2+ lag3 quarters’ consumptions) | 20.5 | 0.67 |
| ARIMA (0,0,0) with exogenous regressors (lag1+ lag2+ lag3+ lag4 quarters’ consumptions) | 24.2 | 1.04 |
| **ICU models** |  |  |
| ARIMA (0,0,0) with exogenous regressors (lag1 semester consumption) | 9.64 | 0.50 |
| ARIMA (0,1,0) with exogenous regressors (lag1 semester consumption) | 10.65 | 0.43 |
| SARIMA (1,0,1) (0,1,0) with exogenous regressors (lag1+ lag2 semesters’ consumptions) | -79.07 | 0.53 |

**Supplementary Figure S1: Hospital logit(resistance) model's residuals**

**Supplementary Figure S2: ICU logit(resistance) model's residuals**

**Supplementary Figure S3: Hospital consumption model's residuals**


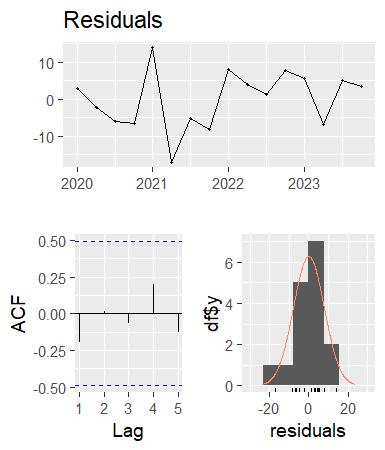


**Supplementary Figure S4: ICU consumption model’s residuals**


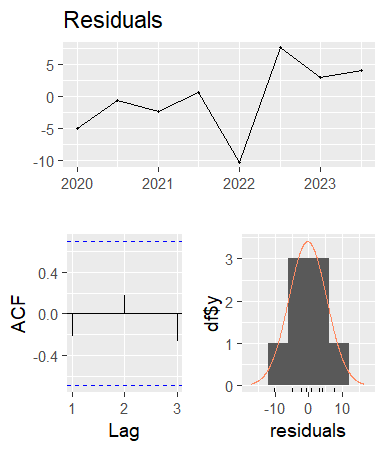


| **Supplementary Table S3: Box-Pierce test results for the four final models** | | |
| --- | --- | --- |
| Model | Chi-squared (df) | *p* |
| Carbapenems consumption |  |  |
| Hospital models |  |  |
| ARIMA (0,0,0) model for consumption trained on data from Q2 2020 to 2023 | 5.78 (10) | 0.833 |
| ICU models |  |  |
| ETS (MAN, damped = True) model for consumption trained on data from S1 2019 to S2 2023 | 2.43 (3) | 0.489 |
| Logit-transformed % Carbapenems resistance |  |  |
| Hospital models |  |  |
| ARIMA (0,1,1) with exogenous regressors (lag1 quarter consumption) | 2.95 (10) | 0.983 |
| ICU models |  |  |
| ARIMA (0,1,0) with exogenous regressors (lag1 semester consumption) | 4.73 (3) | 0.193 |

| **Supplementary Table S4. Forecasted carbapenem resistance in *Klebsiella* spp. at the hospital and ICU levels from 2024 to 2029** | | | | | | | |
| --- | --- | --- | --- | --- | --- | --- | --- |
| **Hospital Model** | | | | **ICU Model** | | | |
| **Quarter** | **Point Forecast** | **80% PI** | **95% PI** | **Semester** | **Point Forecast** | **80% PI** | **95% PI** |
| Q1 2024 | 50.6% | (39.2% to 61.8%) | (33.6% to 67.4%) |  |  |  |  |
| Q2 2024 | 49.8% | (38.5% to 61.1%) | (32.9% to 66.8%) | S1 2024 | 68.1% | (58.3% to 76.6%) | (52.7% to 80.4%) |
| Q3 2024 | 49.8% | (38.5% to 61.2%) | (32.9% to 66.8%) |  |  |  |  |
| Q4 2024 | 49.8% | (38.5% to 61.2%) | (32.9% to 66.8%) | S2 2024 | 71.0% | (57.3% to 81.7%) | (49.4% to 86.0%) |
| Q1 2025 | 49.8% | (38.5% to 61.2%) | (32.9% to 66.8%) |  |  |  |  |
| Q2 2025 | 49.8% | (38.5% to 61.2%) | (32.9% to 66.8%) | S1 2025 | 72.4% | (55.7% to 84.6%) | (46.0% to 89.0%) |
| Q3 2025 | 49.8% | (38.5% to 61.2%) | (32.9% to 66.8%) |  |  |  |  |
| Q4 2025 | 49.8% | (38.5% to 61.2%) | (32.8% to 66.8%) | S2 2025 | 73.7% | (54.5% to 86.8%) | (43.3% to 91.1%) |
| Q1 2026 | 49.8% | (38.5% to 61.2%) | (32.8% to 66.9%) |  |  |  |  |
| Q2 2026 | 49.8% | (38.4% to 61.2%) | (32.8% to 66.9%) | S1 2026 | 74.9% | (53.6% to 88.5%) | (41.1% to 92.7%) |
| Q3 2026 | 49.8% | (38.4% to 61.2%) | (32.8% to 66.9%) |  |  |  |  |
| Q4 2026 | 49.8% | (38.4% to 61.3%) | (32.8% to 66.9%) | S2 2026 | 76.0% | (52.8% to 90.0%) | (39.2% to 94.0%) |
| Q1 2027 | 49.8% | (38.4% to 61.3%) | (32.8% to 66.9%) |  |  |  |  |
| Q2 2027 | 49.8% | (38.4% to 61.3%) | (32.8% to 66.9%) | S1 2027 | 77.1% | (52.2% to 91.2%) | (37.6% to 94.9%) |
| Q3 2027 | 49.8% | (38.4% to 61.3%) | (32.7% to 66.9%) |  |  |  |  |
| Q4 2027 | 49.8% | (38.4% to 61.3%) | (32.7% to 67.0%) | S2 2027 | 78.0% | (51.6% to 92.2%) | (36.1% to 95.7%) |
| Q1 2028 | 49.8% | (38.4% to 61.3%) | (32.7% to 67.0%) |  |  |  |  |
| Q2 2028 | 49.8% | (38.4% to 61.3%) | (32.7% to 67.0%) | S1 2028 | 78.9% | (51.1% to 93.1%) | (34.8% to 96.3%) |
| Q3 2028 | 49.8% | (38.3% to 61.3%) | (32.7% to 67.0%) |  |  |  |  |
| Q4 2028 | 49.8% | (38.3% to 61.3%) | (32.7% to 67.0%) | S2 2028 | 79.7% | (50.7% to 93.8%) | (33.5% to 96.9%) |
| Q1 2029 | 49.8% | (38.3% to 61.3%) | (32.7% to 67.0%) |  |  |  |  |
| Q2 2029 | 49.8% | (38.3% to 61.4%) | (32.6% to 67.1%) | S1 2029 | 80.5% | (50.2% to 94.4%) | (32.4% to 97.3%) |
| Q3 2029 | 49.8% | (38.3% to 61.4%) | (32.6% to 67.1%) |  |  |  |  |
| Q4 2029 | 49.8% | (38.3% to 61.4%) | (32.6% to 67.1%) | S2 2029 | 81.2% | (49.8% to 95.0%) | (31.3% to 97.6%) |

Point forecasts and corresponding 80% and 95% prediction intervals (PI) for carbapenem resistance (%) are shown for the hospital and ICU models. Hospital-level forecasts are presented quarterly and were derived from an ARIMA model using carbapenem consumption expressed as defined daily doses per 1,000 patient-days (DDD/1000PD). ICU-level forecasts are presented by semester and were derived from an exponential smoothing model using carbapenem consumption expressed as days of therapy per 1,000 patient-days (DOT/1000PD). Differences in time aggregation and consumption metrics reflect data availability and clinical context; forecasts should therefore be interpreted within each setting independently


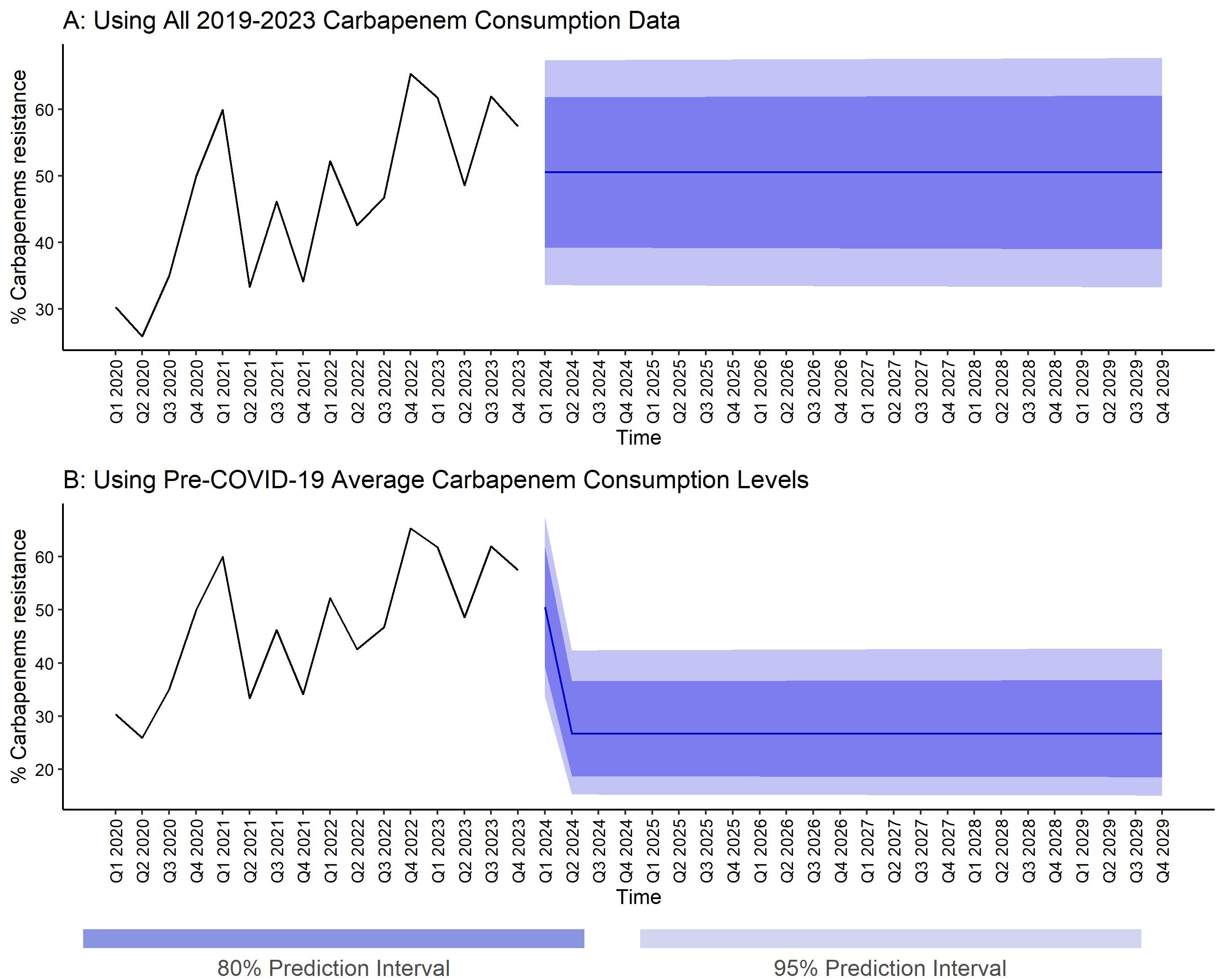


**Supplementary Figure S5**

| **Supplementary Table S5. Sensitivity analysis of hospital-level carbapenem resistance forecasts under alternative carbapenem consumption training strategies** | | | | | | |
| --- | --- | --- | --- | --- | --- | --- |
| **Quarter** | **Forecasts using 2019 to 2023 carbapenem consumption data** | | | **Forecasts using the average pre-COVID-19 carbapenem consumption level** | | |
|  | **Point Forecast** | **80% PI** | **95% PI** | **Point Forecast** | **80% PI** | **95% PI** |
| Q1 2024 | 50.6% | (39.2% to 61.8%) | (33.6% to 67.4%) | 50.6% | (39.2% to 61.8%) | (33.6% to 67.4%) |
| Q2 2024 | 50.6% | (39.2% to 61.8%) | (33.6% to 67.4%) | 26.7% | (18.7% to 36.6%) | (15.2% to 42.4%) |
| Q3 2024 | 50.6% | (39.2% to 61.8%) | (33.6% to 67.4%) | 26.7% | (18.7% to 36.6%) | (15.2% to 42.4%) |
| Q4 2024 | 50.6% | (39.2% to 61.9%) | (33.6% to 67.4%) | 26.7% | (18.6% to 36.6%) | (15.2% to 42.4%) |
| Q1 2025 | 50.6% | (39.2% to 61.9%) | (33.5% to 67.4%) | 26.7% | (18.6% to 36.6%) | (15.2% to 42.4%) |
| Q2 2025 | 50.6% | (39.2% to 61.9%) | (33.5% to 67.5%) | 26.7% | (18.6% to 36.6%) | (15.2% to 42.4%) |
| Q3 2025 | 50.6% | (39.2% to 61.9%) | (33.5% to 67.5%) | 26.7% | (18.6% to 36.6%) | (15.2% to 42.4%) |
| Q4 2025 | 50.6% | (39.2% to 61.9%) | (33.5% to 67.5%) | 26.7% | (18.6% to 36.6%) | (15.2% to 42.5%) |
| Q1 2026 | 50.6% | (39.1% to 61.9%) | (33.5% to 67.5%) | 26.7% | (18.6% to 36.6%) | (15.2% to 42.5%) |
| Q2 2026 | 50.6% | (39.1% to 61.9%) | (33.5% to 67.5%) | 26.7% | (18.6% to 36.6%) | (15.2% to 42.5%) |
| Q3 2026 | 50.6% | (39.1% to 61.9%) | (33.4% to 67.5%) | 26.7% | (18.6% to 36.6%) | (15.2% to 42.5%) |
| Q4 2026 | 50.6% | (39.1% to 61.9%) | (33.4% to 67.5%) | 26.7% | (18.6% to 36.7%) | (15.2% to 42.5%) |
| Q1 2027 | 50.6% | (39.1% to 61.9%) | (33.4% to 67.6%) | 26.7% | (18.6% to 36.7%) | (15.1% to 42.5%) |
| Q2 2027 | 50.6% | (39.1% to 62.0%) | (33.4% to 67.6%) | 26.7% | (18.6% to 36.7%) | (15.1% to 42.6%) |
| Q3 2027 | 50.6% | (39.1% to 62.0%) | (33.4% to 67.6%) | 26.7% | (18.6% to 36.7%) | (15.1% to 42.6%) |
| Q4 2027 | 50.6% | (39.1% to 62.0%) | (33.4% to 67.6%) | 26.7% | (18.6% to 36.7%) | (15.1% to 42.6%) |
| Q1 2028 | 50.6% | (39.1% to 62.0%) | (33.4% to 67.6%) | 26.7% | (18.6% to 36.7%) | (15.1% to 42.6%) |
| Q2 2028 | 50.6% | (39.0% to 62.0%) | (33.3% to 67.6%) | 26.7% | (18.6% to 36.7%) | (15.1% to 42.6%) |
| Q3 2028 | 50.6% | (39.0% to 62.0%) | (33.3% to 67.6%) | 26.7% | (18.5% to 36.7%) | (15.1% to 42.6%) |
| Q4 2028 | 50.6% | (39.0% to 62.0%) | (33.3% to 67.7%) | 26.7% | (18.5% to 36.7%) | (15.1% to 42.7%) |
| Q1 2029 | 50.6% | (39.0% to 62.0%) | (33.3% to 67.7%) | 26.7% | (18.5% to 36.7%) | (15.1% to 42.7%) |
| Q2 2029 | 50.6% | (39.0% to 62.0%) | (33.3% to 67.7%) | 26.7% | (18.5% to 36.8%) | (15.1% to 42.7%) |
| Q3 2029 | 50.6% | (39.0% to 62.1%) | (33.3% to 67.7%) | 26.7% | (18.5% to 36.8%) | (15.1% to 42.7%) |
| Q4 2029 | 50.6% | (39.0% to 62.1%) | (33.3% to 67.7%) | 26.7% | (18.5% to 36.8%) | (15.0% to 42.7%) |

Quarterly point forecasts and corresponding 80% and 95% prediction intervals (PI) for carbapenem resistance (%) derived from hospital-level models trained using two alternative carbapenem consumption inputs: (i) the full 2019–2023 carbapenem consumption time series and (ii) the average pre-COVID-19 carbapenem consumption level. This sensitivity analysis was conducted to assess the robustness of resistance projections to different assumptions regarding the consumption training period. Forecasts are presented for comparison with the primary post-COVID-19 hospital model.
